# Perioperative and 6-month outcomes of UNSES versus UBE for lumbar disc herniation with standardized annular suture

**DOI:** 10.3389/fsurg.2026.1858219

**Published:** 2026-09-11

**Authors:** Jie Cang, Chengjia Wang, Xianwen Yan, Dapeng Li, En Song

**Affiliations:** 1 Department of Spine Surgery, Affiliated Hospital of Jiangsu University, Zhenjiang, Jiangsu, China; 2 Department of Sports Medicine, The First Affiliated Hospital of Kunming Medical University, Kunming, Yunnan, China

**Keywords:** clinical efficacy, endoscopic discectomy, lumbar disc herniation, unilateral biportal endoscopy, uniportal non-coaxial spinal endoscopic surgery

## Abstract

**Background:**

Unilateral biportal endoscopy (UBE) and uniportal non-coaxial spinal endoscopic surgery (UNSES) are mainstream minimally invasive approaches for lumbar disc herniation (LDH). Few studies have systematically compared their perioperative and medium-term clinical outcomes when all patients receive standardized annulus fibrosus (AF) suture. This retrospective cohort study aimed to compare perioperative indicators and 6-month functional, radiological outcomes between UNSES and UBE in patients with LDH treated with identical annular suture protocols.

**Methods:**

The clinical data of 69 patients with LDH admitted between January 2024 and January 2025 were retrospectively analyzed. The patients were divided into the UNSES group (33 cases) and UBE group (36 cases), both undergoing annulus fibrosus suture. Surgical-related indicators, clinical outcomes [low back pain visual analog scale (VAS), radicular leg pain VAS, and Oswestry Disability Index (ODI) scores], radiological parameters (annulus fibrosus closure rate), modified MacNab excellent and good rate, and complications were compared.

**Results:**

The UNSES group of patients had a significantly shorter operation time, less intraoperative blood loss, and lower postoperative C-reactive protein increment than their UBE counterparts (all *P* < 0.05). No significant differences were found in fluoroscopy times or hospital stay (both *P* > 0.05). Both patient groups showed significant improvements in VAS and ODI scores postoperatively (all *P* < 0.05). At 3 and 6 months postoperatively, the UNSES group had significantly lower low back pain VAS and radicular leg pain VAS scores, while the UBE group had a lower ODI score and higher annulus fibrosus closure rate at 6 months (all *P* < 0.05). The modified MacNab excellent and good rate (93.9% vs. 88.9%) and complication rate (3.03% vs. 8.33%) showed no significant differences (both *P* > 0.05).

**Conclusion:**

Both UNSES and UBE achieve safe and effective short-term efficacy for LDH when combined with standardized annular suture. UNSES is associated with milder perioperative surgical trauma and sustained lower low back and radicular leg pain within 6 months, whereas UBE yields better 6-month lumbar function scores. Extended 1–2-year longitudinal follow-up and controlled trials without annular repair are required to further investigate whether AF suturing exerts long-term protective effects against disc degeneration and reherniation, which cannot be confirmed by our 6-month short-term data.

## 1 Introduction

Lumbar disc herniation (LDH) is a common clinical spinal degenerative disease, with low back pain and radiating pain of lower limbs as the typical symptoms (1). More than half of the patients require long-term clinical intervention because of the recurrence of their conditions (2, 3). Previous studies have indicated that annulus fibrosus (AF) suture may lower postoperative reherniation risk and retard intervertebral disc degeneration; however, such claims need verification through trials with a non-suture control group, which our study does not contain. With the development of minimally invasive techniques, unilateral biportal endoscopy (UBE) and uniportal non-coaxial spinal endoscopic surgery (UNSES) have become mainstream minimally invasive procedures for LDH treatment (4). UBE adopts a dual-channel design with broad operative vision and flexible manipulation, yet dual-tract establishment may aggravate paraspinal soft tissue injury (5). UNSES allows a parallel operation of endoscope and instruments through a single incision, leading to milder soft tissue trauma but limited intraspinal operating space (6). To date, few systematic head-to-head comparisons have evaluated perioperative metrics, complication profiles, and short-term follow-up results between UBE and UNSES when standardized annulus fibrosus suture is uniformly performed on all subjects. Existing comparative papers rarely adopt a consistent annular repair scheme for both groups, making it difficult to isolate the intrinsic technical differences and provide clinical evidence between the two endoscopic modalities. All 69 single-segment LDH patients enrolled in this retrospective cohort received identical standardized annulus fibrosus suture regardless of the assigned endoscopic group. The present study aimed to compare the short-term perioperative and 6-month clinical and radiological outcomes of UNSES and UBE in patients with single-segment lumbar disc herniation who all received standardized annulus fibrosus suture, so as to supply evidence for individualized endoscopic surgical selection in clinical practice.

## 2 Materials and methods

### 2.1 General data

A retrospective cohort study was conducted on 69 patients with LDH admitted between January 2024 and January 2025. Patients were divided into the UNSES group (33 cases, treated with UNSES combined with manual interrupted annulus fibrosus suture) and the UBE group (36 cases, treated with UBE combined with the same suture technique). This retrospective clinical cohort study was approved by the Medical Ethics Committee of our hospital (Ethics No.: KY2025K1203). All patient clinical data were fully anonymized before analysis. The ethics committee waived the requirement for written informed consent in compliance with the Declaration of Helsinki, and all original medical records were kept strictly confidential without any identifiable personal information. All patients completed 6 months of outpatient follow-up without loss to follow-up.

Inclusion Criteria: (1) Diagnosis of LDH confirmed by MRI or CT, with consistent protruding segments and clinical symptoms; (2) Failure of standardized conservative treatment for 3 months; (3) Single-segment disc herniation (L2/3-L5/S1) with protrusion type being inclusive or simple protrusion (excluding sequestered type); (4) Annulus fibrosus tear diameter ≤4 mm (evaluated by preoperative MRI or intraoperative endoscopy), suitable for manual interrupted suture; (5) Complete clinical data and postoperative follow-up ≥6 months.

Exclusion Criteria: (1) Multiple-segment herniation (≥2 segments with disc herniation ≥5 mm), lumbar spinal stenosis, spondylolisthesis, fracture, or other comorbidities; (2) Previous spinal surgery history; (3) Coagulopathy, infectious diseases, or other severe underlying diseases; (4) Annulus fibrosus tear >4 mm that cannot be effectively sutured; (5) Lumbar instability (vertebral slip >3 mm on preoperative dynamic X-ray); (6) Loss to follow-up or incomplete data.

**Figure 1:**
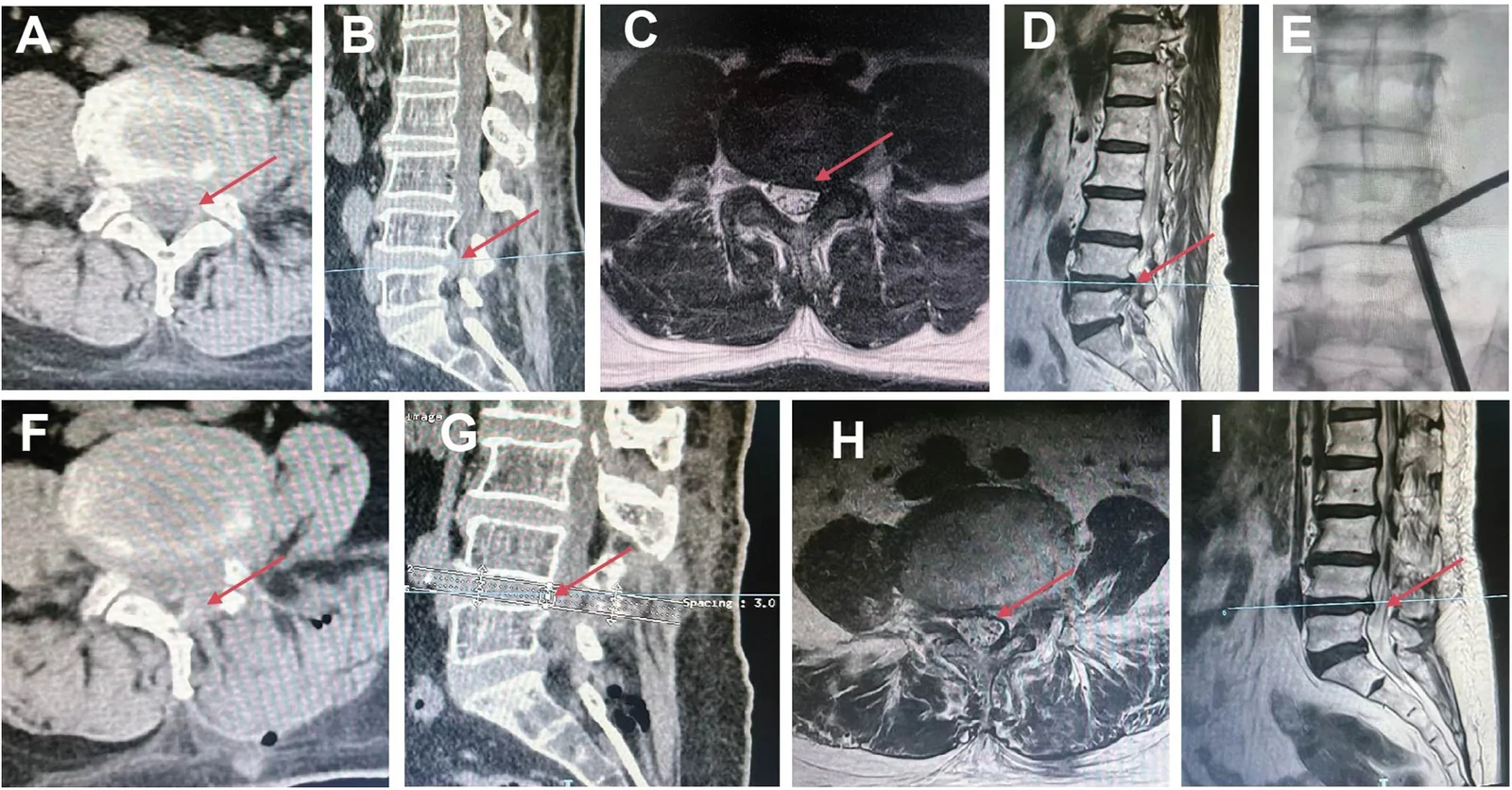
Preoperative and postoperative lumbar imaging sequences of representative single-segment lumbar disc herniation patients. The red arrows in all subgraphs mark the target herniated disc segment and surgical repair area. **(A,B)** Axial and sagittal preoperative lumbar CT demonstrating intervertebral disc protrusion; **(C,D)** Axial and sagittal preoperative T2-weighted MRI confirming the herniated mass compressing the neural canal; **(E)** Intraoperative fluoroscopic localization radiograph for the target intervertebral space; **(F,G)** Postoperative axial and sagittal CT showing adequate bony decompression of the spinal canal; **(H,I)** Postoperative 6-month axial and sagittal T2-weighted MRI revealing continuous fibrous tissue coverage of the sutured annular defect.

All enrolled patients received either UNSES or UBE endoscopic discectomy within the same consecutive study period. All surgical procedures were performed by a unified spine surgery team consisting of three attending surgeons, each of whom had accumulated more than 200 cases of single-channel endoscopy and over 200 cases of biportal UBE surgery before the start of patient enrollment, with balanced operative experience for both techniques. Surgical modality selection followed combined objective imaging criteria and clinical judgment rather than random assignment. Preoperative MRI and CT were reviewed to comprehensively evaluate key anatomical factors: lumbar canal stenosis grade, herniation location (central/lateral/foraminal), maximum size of annulus fibrosus defect, spinal canal volume, and patient body habitus (BMI, paraspinal muscle thickness). For patients with large annular tears, severe lateral recess stenosis, or high BMI, UBE was preferentially adopted for its broad operative visual field. UNSES was primarily selected for patients with small-moderate herniation, mild stenosis, and thin paraspinal muscles, aiming to minimize soft tissue trauma. The final surgical decision was confirmed by the attending surgeon based on integrated imaging and patient conditions. No fixed rigid grouping protocol was applied, and surgeon clinical preference served only as an auxiliary factor after a comprehensive anatomical assessment. Preoperative baseline data were systematically collected for all participants, including demographic data (age, sex, BMI, affected lumbar segment), clinical manifestations [symptom duration, presence of neurological deficit, preoperative pain visual analog scale (VAS) score, preoperative Oswestry Disability Index (ODI) score], and imaging features (herniation type, herniation location, annulus fibrosus tear location). Morphological classification of intervertebral disc herniation and annular tear location was determined by reviewing preoperative sagittal and axial lumbar MRI. There was no significant difference in preoperative baseline data between the two groups (*P* > 0.05), showing comparability (Table 1). Preoperative imaging examinations included preoperative lumbar spine CT scan (Figures 1A,B), preoperative lumbar spine MRI scan (Figures 1C,D), intraoperative localization map (Figure 1E), postoperative lumbar spine CT scan (Figures 1F,G), and postoperative lumbar spine MRI scan (Figures 1H,I).

**Table 1:** Comparison of preoperative baseline data ($\bar{x}$ ± *s*, *n*).

Items	UNSES group (*n* = 33)	UBE group (*n* = 36)	*χ*^2^/*t*-value	*P*-value
Age (years)	49.4±15.4	51.8±13.9	0.68	0.501
Gender (male/female)	18/15	14/22	1.81	0.179
BMI (kg/m^2^)	24.0±1.5	23.7±1.0	0.97	0.335
Symptom duration (months)	12.6±4.2	13.2±4.3	−0.53	0.600
Preoperative pain VAS score	7.12 ± 0.96	7.08 ± 0.84	0.18	0.855
Preoperative ODI score (%)	65.88 ± 6.24	63.94 ± 6.92	0.07	0.944
Neurological deficit			0.07	0.788
Yes	18	20		
No	15	16		
Affected segment			3.38	0.338
L2/3	1	1		
L3/4	1	4		
L4/5	19	14		
L5/S1	12	17		
Herniation type			0.20	0.907
Protrusion	14	16		
Extrusion	13	14		
Sequestration	6	6		
Herniation location			0.25	0.883
Central	11	13		
Lateral	16	17		
Foraminal	6	6		
Annular tear location			0.31	0.858
Central	10	12		
Lateral recess	17	18		
Foraminal	6	6		

### 2.2 Surgical methods

#### 2.2.1 UNSES group

**Figure 2:**
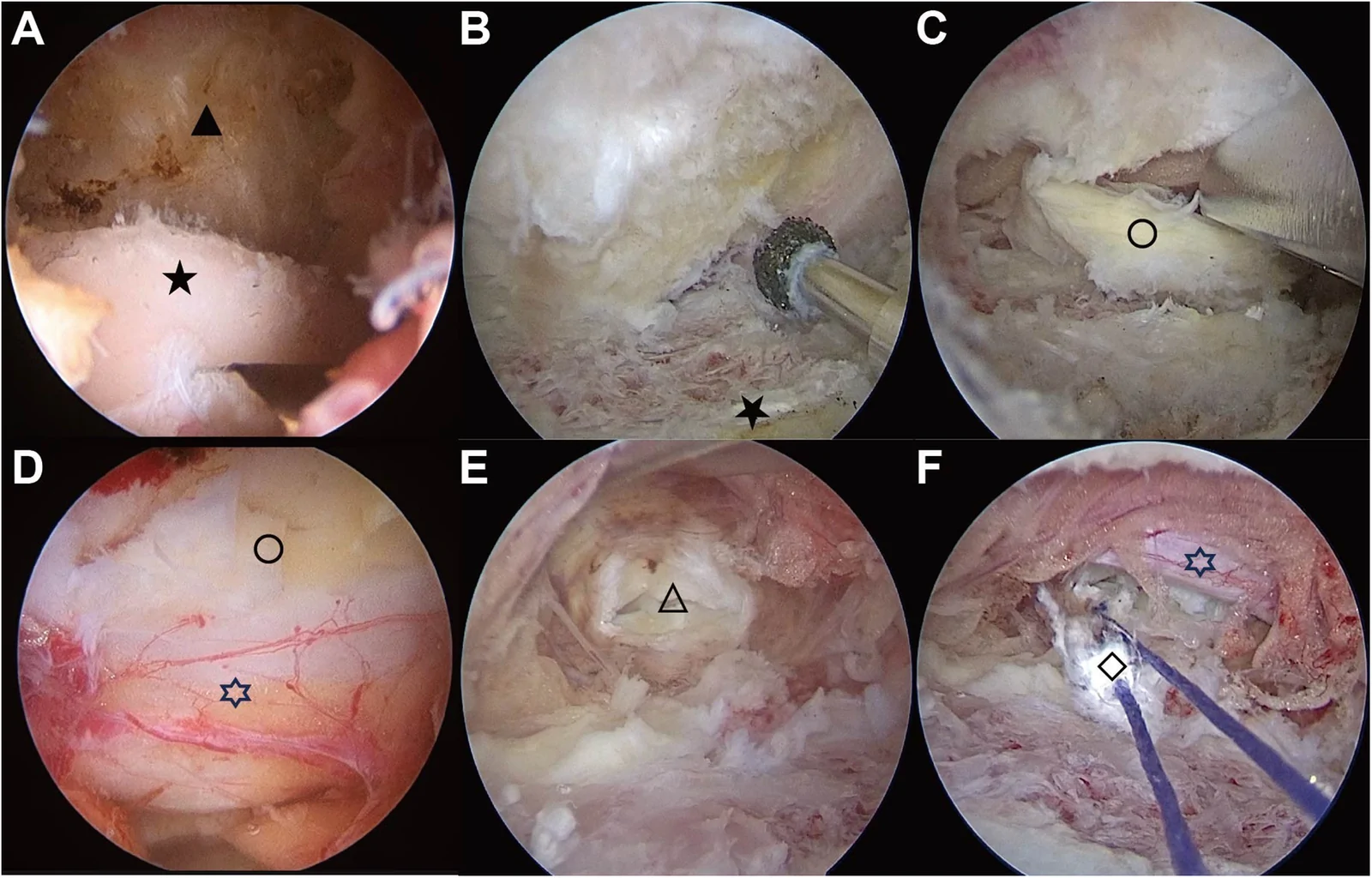
A step-by-step intraoperative endoscopic workflow of the UNSES technique, with standardized marker symbols defined as follows: ★ inferior margin of upper lamina, ▴ interlaminar space, ◯ ligamentum flavum, ⋆ dural sac and epidural venous plexus, △ enlarged intervertebral foramen, ◇ sutured annulus fibrosus defect. **(A)** Initial endoscopic exposure of bony laminar structures and herniated disc tissue. **(B)** A bony resection of lamina and articular process osteophytes with a high-speed drill. **(C)** The separation and resection of the ligamentum flavum to expose deep spinal canal structures. **(D)** A full visualization of the dural sac and epidural vascular plexus after decompression. **(E)** Foraminoplasty to release the exiting nerve root and confirm free nerve root mobility. **(F)** An interrupted suture repair of the annular tear using absorbable sutures.

Taking left L5/S1 LDH as an example, the patients were placed in the prone position, and a silicone stent was placed under the abdomen to maintain posture after general anesthesia. The L5/S1 intervertebral space was localized by C-arm fluoroscopy, and the reference line was marked on the body surface. A 1.5-cm longitudinal incision was made along the guide wire after puncturing the lateral aspect of the lamina near the proximal articular process through the medial pedicle of the left vertebra. The channel was expanded with dilators after sequential incision of tissues, and the working cannula was inserted to connect the spinal endoscopy system. Under endoscopy, structures such as the lower edge of the lamina and ligamentum flavum were identified. The lamina window was enlarged with a drill, the ligamentum flavum was stripped and dissected to expose the spinal canal, and the nerve root was released until it regained relaxation. Residual nucleus pulposus tissue in the spinal canal was checked (Figure 2).

Subsequently, identical 4-0 absorbable polyglactin suture and dedicated spinal endoscopic annular suture forceps (uniform for both UNSES and UBE groups) were used for interrupted repair. Three standardized suture patterns were selected based on the morphological width and shape of the annular tear: a parallel double-needle suture for narrow linear lacerations, Y-shaped suture for small triangular defects, and fence-type suture for wide irregular tears. A unified stitch standard was applied for all patients: 1–2 stitches were placed on each edge of the tear, forming a total of 2–4 stitches per annular defect. Because of the single narrow working channel inherent to UNSES, only miniaturized curved suture forceps could be used; repeated fine adjustment of instrument angles was required to avoid blocking the endoscopic visual field during needle penetration and knot tying. After completing interrupted suturing, a full endoscopic reinspection was performed to confirm satisfactory closure under the following unified intraoperative criteria: no persistent gap on the sutured annulus, no residual herniated nucleus pulposus tissue protruding from the tear, and no obvious irrigation fluid leakage at the repair site, as well as no suture entanglement or compression on traversing/exiting nerve roots. Finally, the temporarily suspended ligamentum flavum was reset to cover the neural structures. The surgical field was fully irrigated, a drainage tube was indwelled, and the incision was sutured layer by layer.

#### 2.2.2 UBE group

Preoperative preparation and intervertebral space localization were consistent with the UNSES group. Taking the intersection of the midline of the intervertebral space and the inner edge of the pedicle as a reference, the observation port was located approximately 1–1.5 cm above, and the surgical port was 1–1.5 cm below, with a distance of 2–3 cm between the two ports to avoid mutual interference during operation. An intervertebral foramen endoscope was inserted through the cephalic observation port to form a clear visual path, and the caudal surgical port served as a channel for surgical instruments to facilitate the access and flexible control of various decompression and repair instruments.

*Procedure*: The skin, subcutaneous tissue, and deep fascia of the two incisions were incised sequentially. A muscle separator was used to penetrate the paraspinal muscles to the lamina surface, and the soft tissues attached to the lamina were dissected to expose the bony structure. On the surgical channel side, a radiofrequency scalpel was used to precisely dissect the ligamentum flavum and residual soft tissues on the lamina surface to avoid injury to surrounding blood vessels and nerves. Subsequently, laminotomy and decompression were performed to completely remove osteophytes and thickened ligamentum flavum, fully exposing the dural sac and nerve roots. The subsequent annulus fibrosus suture was the same as that in the UNSES group. Notably, the dual independent observation and working channels of UBE bring distinct advantages for annular repair compared with single-channel UNSES: the endoscope can be fixed at a stable viewing angle without frequent displacement during suturing, and conventional-sized large-curved suture forceps can be freely manipulated with multiangle needle rotation, which greatly simplifies edge alignment for wide or irregular annular tears. The same suture material, instruments, stitch quantity standard, and intraoperative satisfactory closure evaluation criteria as the UNSES group were strictly implemented to guarantee consistent annular repair quality between the two cohorts.

*Surgical completion criteria*: (1) Adequate nerve root decompression, with spontaneous nerve root pulsation and negative traction test result; (2) Satisfactory closure of the annulus fibrosus defect without obvious leakage; (3) No active bleeding in the surgical field; (4) No residual instruments or injury to the nerves/dural sac.

#### 2.2.3 Unified perioperative management protocol

All patients in the UNSES and UBE groups received identical standardized perioperative regimens to rule out confounding bias from inconsistent clinical interventions. All participants completed unified preoperative laboratory and lumbar imaging examinations within 3 days before surgery, with 3 days of preoperative oral celecoxib for preemptive analgesia and standardized preoperative functional exercise guidance. Tracheal intubation general anesthesia was adopted uniformly for all operations, with consistent induction, maintenance, and intraoperative fluid management protocols.

A unified multimodal analgesia scheme was applied postoperatively: intravenous flurbiprofen axetil was used within 48 h after surgery, followed by 2 weeks of oral celecoxib after discharge, and tramadol injection was provided as rescue analgesia only when VAS ≥ 7. Subcutaneous low-molecular-weight heparin calcium was administered for 7 days starting 12 h postoperatively for venous thrombosis prophylaxis in patients with normal coagulation function. Perioperative cefuroxime sodium was used for 24 h as prophylactic antibiotics without long-term postoperative anti-infection treatment. Uniform rehabilitation standards were implemented for all patients. Drainage tubes were removed when daily drainage volume was less than 50 mL. Patients ambulated with a rigid lumbar brace on postoperative day 2, performed standardized straight leg raising training twice daily within the first 2 weeks, and started lumbar core strengthening exercises 1 month after discharge, with identical training intensity and frequency across both groups.

### 2.3 Outcome measures

(1) *Surgical-related indicators*: Operation time, calculated total intraoperative blood loss, intraoperative fluoroscopy times, length of hospital stay, and postoperative C-reactive protein (CRP) increment (unit: mg/L). Calculated blood loss was estimated through the hemoglobin difference method: Blood loss (mL) = (preoperative Hb−postoperative Hb)/preoperative Hb × total blood volume × 0.8. The coefficient of 0.8 was adopted to correct interstitial fluid dilution after the operation; the standard adult blood volume was defined as 70 mL/kg. Uniform detection timing was implemented for all patients: Preoperative venous hemoglobin was tested within 24 h before the surgery, and postoperative hemoglobin was measured at 06:00 on postoperative day 1 (POD 1). Unified intraoperative fluid infusion regimens were applied to all patients to reduce intergroup hemodilution bias. Serum CRP was detected at two fixed time points: baseline measurement within 24 h preoperatively and postoperative detection on the morning of postoperative day 2 (POD 2). CRP increment was calculated as postoperative CRP value minus preoperative baseline CRP value, with the unit uniformly defined as mg/L. (2) Low back pain VAS and radicular leg pain VAS scores (0–10 points; higher scores represent more severe pain) were separately documented before surgery, 3 and 6 months postoperatively. The ODI (range 0%–100%, with higher values indicating worse lumbar dysfunction) was assessed at the identical three time points to quantify patients' functional impairment. (3) Therapeutic effect: Modified MacNab criteria (Excellent, Good, Fair, Poor) were used to evaluate efficacy at 6 months postoperatively. (4) *Complications*: Incidence of hematoma, infection, nerve injury, dural sac tear, and other complications, as well as treatment and prognosis. (5) *Radiological evaluation of annulus fibrosus*: All patients received uniform manual interrupted annular repair with consistent suture specifications regardless of surgical group before a postoperative MRI evaluation of AF healing. Lumbar spine T2-weighted MRI obtained at 6 months postoperatively was used to evaluate the healing status of intraoperative annular defects, with unified objective imaging criteria adopted for grading AF healing; all MRI scans were independently reviewed by two senior spinal surgeons who remained blinded to patient group allocation before formal image assessment, and interobserver reliability of the AF grading system was validated with a Kappa value of 0.89, which represented excellent inter-rater agreement. The standardized morphological definitions were as follows: complete AF closure was defined as continuous low-signal fibrous tissue fully covering the original annular defect without residual high-signal fluid cleft or recurrent nucleus pulposus herniation on sagittal and axial sequences; partial closure referred to partial coverage of the tear by fibrous tissue with a tiny residual high-signal gap at the defect site; incomplete closure indicated persistent wide high-signal separation at the tear accompanied by obvious discontinuity of the annulus fibrosus. The primary radiological endpoint of this analysis was the complete AF closure rate, calculated as the number of patients with complete closure divided by the total sample size of each group.

### 2.4 Statistical analysis

IBM SPSS 22.0 statistical software was used for data analysis. Repeated-measures analysis of variance (ANOVA) was used for comparing repeated measurement data between the two groups. Enumeration data (gender, affected segment, therapeutic effect) were expressed as constituent ratios or rates, and intergroup comparisons were performed using the *χ*^2^ test. Ranked data were analyzed using the Mann–Whitney *U* test. A value of *P* < 0.05 was considered statistically significant. When significant group-time interaction was detected through repeated-measures ANOVA, *post hoc* pairwise intergroup comparisons at each independent time point were performed with Bonferroni correction to adjust for multiple testing bias. Mean between-group differences and corresponding 95% confidence intervals together with exact *P*-values were calculated and documented for each follow-up time point.

## 3 Results

### 3.1 Comparison of surgical-related indicators

The operation time, intraoperative blood loss, and postoperative CRP increment in the UNSES group were significantly lower than those in the UBE group (*P* < 0.05). There were no significant differences in intraoperative fluoroscopy times or length of hospital stay between the two groups (*P* > 0.05). The near-identical average intraoperative fluoroscopy frequency between the two groups can be attributed to the unified C-arm fluoroscopy localization protocol adopted by the same surgical team for all single-segment LDH patients. Fluoroscopy was used only once to confirm target intervertebral space position before skin incision; no additional fluoroscopic scanning was required during intraspinal decompression, nucleus pulposus resection, or annulus fibrosus suturing steps, leading to concentrated fluoroscopy count values and small standard deviations in both cohorts (Table 2).

**Table 2:** Comparison of surgical indicators ($\bar{x}$ ± s).

Items	UNSES group (*n* = 33)	UBE group (*n* = 36)	*t*-Value	*P*-value
Operation time (min)	104.7 ± 25.2	119.4 ± 17.5	2.79	0.007
Preoperative Hb (g/L)	142.5 ± 14.1	139.3 ± 14.8	0.92	0.360
Postoperative Hb (g/L)	132.5 ± 13.4	126.0 ± 12.9	2.05	0.044
Intraoperative blood loss (mL)	322.3 ± 97.3	435.0 ± 93.5	4.90	<0.001
Intraoperative fluoroscopy times (*n*)	4.15 ± 0.62	4.28 ± 0.71	0.86	0.412
Length of hospital stay (day)	9.3 ± 1.5	10.2 ± 3.7	1.34	0.185
Postoperative CRP increment (mg/L)	22.36 ± 1.18	23.96 ± 1.99	4.10	<0.001

### 3.2 Comparison of postoperative VAS score, ODI score, and 6-month annulus fibrosus closure rate

Repeated-measures ANOVA detected significant time, intergroup, and group-time interaction effects for low back pain VAS, radicular leg pain VAS, and ODI scores (all overall *P* < 0.05). Both groups achieved an obvious relief of back and radicular leg pain, as well as marked improvement in lumbar function at 3 and 6 months after surgery. All pairwise intergroup comparisons at each time point were performed using Bonferroni correction, and detailed statistical data, including mean differences, 95% Cis, and adjusted *P*-values, are presented in Table 3.

**Table 3:** Comparison of low back VAS, radicular leg VAS, and ODI scores ($\bar{x}$ ± s).

Items	Time points	UNSES (*n* = 33)	UBE (*n* = 36)	Mean diff. (95% CI)	*P* (Bonferroni)	Overall *F*	*P*-value
Low back pain VAS score	Preoperative	7.12 ± 0.96	7.08 ± 0.84	−0.04 (−0.42, 0.34)	0.831	5.23 (int.)	0.024
	3 months postop	2.12 ± 0.65	2.56 ± 0.56	0.44 (0.17, 0.71)	0.002	712.65 (time)	<0.001
	6 months postop	1.76 ± 0.50	2.17 ± 0.56	0.41 (0.16, 0.66)	0.003	6.18 (group)	0.015
Radicular leg pain VAS score	Preoperative	6.97 ± 1.01	6.91 ± 0.95	−0.06 (−0.43, 0.31)	0.742	5.41 (int.)	0.021
	3 months postop	1.98 ± 0.61	2.43 ± 0.53	0.45 (0.19, 0.71)	0.002	756.32 (time)	<0.001
	6 months postop	1.62 ± 0.48	2.08 ± 0.54	0.46 (0.20, 0.72)	0.002	6.35 (group)	0.013
ODI score (%)	Preoperative	65.88 ± 6.24	63.94 ± 6.92	−1.94 (−4.72, 0.84)	0.169	4.17 (int.)	0.042
	3 months postop	19.79 ± 4.07	18.64 ± 3.37	−1.15 (−2.81, 0.51)	0.173	1,985.32 (time)	<0.001
	6 months postop	16.03 ± 4.14	13.86 ± 2.68	−2.17 (−3.72, −0.62)	0.007	5.89 (group)	0.018

Mean diff., mean between-group difference; CI, confidence interval; All *P*-values were adjusted by using Bonferroni correction for multiple *post hoc* pairwise comparisons. int., interaction effect; time, time main effect; group, intergroup main effect; VAS, visual analog scale; ODI, Oswestry Disability Index.

At preoperative baseline, there were no significant between-group differences in low back pain, radicular leg pain, and ODI scores, demonstrating comparable baseline pain and functional status. At 3 and 6 months postoperatively, patients in the UNSES group had significantly milder low back pain and radicular leg pain than those receiving UBE. No intergroup difference in the ODI score was observed at the 3-month follow-up, whereas the UBE group exhibited significantly better lumbar functional performance at the 6-month time point.

For the 6-month postoperative lumbar MRI follow-up, two senior spine surgeons blinded to patient grouping independently assessed AF closure using standardized T2-weighted MRI criteria, with all related statistical outcomes listed in Table 4. The complete AF closure rate reached 87.9% (29 out of 33 patients) in the UNSES group and 91.7% (33 out of 36 patients) in the UBE group; partial closure was observed in three patients with UNSES and two patients with UBE, while incomplete closure occurred in one patient with UNSES and one patient with UBE. An intergroup comparison demonstrated a statistically significant difference in complete AF closure rate (*χ*^2^ = 4.02, *P* = 0.045). All AF closure grades were evaluated on unified 6-month postoperative T2-weighted MRI under double-blinded review, and interobserver consistency (Kappa = 0.89) was verified in advance to reduce subjective judgment bias. The broader dual-channel visual field inherent to UBE may allow full visualization of annular lacerations and more accurate edge approximation during suturing, which could partially explain the slightly higher complete AF closure rate observed in the UBE cohort compared with single-channel UNSES.

**Table 4:** Six-month annulus fibrosus closure status (*n*, %).

Items	UNSES group (*n* = 33)	UBE group (*n* = 36)	*χ*^2^ value	*P*-value
Complete AF closure	29 (87.9)	33 (91.7)	4.02	0.045
Partial AF closure	3 (9.1)	2 (5.6)		
Incomplete AF closure	1 (3.0)	1 (2.8)		

AF, annulus fibrosus; *n*, number of patients. Closure status was uniformly judged on postoperative 6-month T2-weighted MRI by two blinded senior spine surgeons according to standardized imaging criteria.

### 3.3 Therapeutic effect and complications

At the final follow-up, the excellent and good rate of modified MacNab criteria was 93.9% (25 Excellent, six Good, one Fair, one Poor) in the UNSES group and 88.9% (23 Excellent, nine Good, two Fair, two Poor) in the UBE group, with no significant intergroup difference (*χ*^2^ = 1.18, *P* = 0.76).

*Complications*: One case (3.03%) of infection occurred in the UNSES group, which was cured with cefuroxime anti-infective treatment for 1 week, with normal body temperature and good incision healing. Three cases (8.33%) of complications occurred in the UBE group: one case of hematoma (absorbed after 1 week of postoperative compression bandaging), one case of infection (cured after anti-infective treatment), and one case of dural sac tear (repaired intraoperatively, no cerebrospinal fluid leakage after 2 weeks of bed rest). There was no significant difference in the complication rate between the two groups (*P* = 0.52) (Table 5).

**Table 5:** Clinical efficacy and complications (*n*, %).

Items	UNSES group (*n* = 33)	UBE group (*n* = 36)	*χ*^2^ value	*P*-value
Therapeutic effect			1.18	0.760
Excellent	25 (75.8)	23 (63.9)		
Good	6 (18.2)	9 (25.0)		
Fair	1 (3.0)	2 (5.6)		
Poor	1 (3.0)	2 (5.6)		
Complications			–	0.520
Hematoma	0 (0.0)	1 (2.8)		
Infection	1 (3.0)	1 (2.8)		
Nerve injury	0 (0.0)	0 (0.0)		
Dural sac tear	0 (0.0)	1 (2.8)		
Total complication rate	1 (3.0)	3(8.3)	–	0.520

## 4 Discussion

This retrospective cohort compared perioperative indicators, 6-month clinical and radiological outcomes, and complication profiles between UNSES and UBE, with standardized manual interrupted AF suture performed uniformly across all patients to eliminate confounding differences in annular management. Overall, both endoscopic modalities effectively relieved pain and improved lumbar function with acceptable safety, while distinct technical differences existed in perioperative trauma. UNSES presents prominent perioperative advantages, which are attributed to its single-channel design. Endoscope and instruments operate in parallel through one single incision, eliminating the need for two separate working tracts. This reduces paraspinal muscle dissection and cuts down the time spent in adjusting instruments, leading to shorter operative durations (7). In this study, the intraoperative blood loss in the UNSES group was significantly lower than that in the UBE group, which was closely related to the role of precise hemostasis under direct vision and annulus fibrosus suture technique in the control of intervertebral space bleeding under a single channel of UNSES (8), while the insertion and operation of UBE double channels may increase the risk of injury to the paraspinal muscles and epidural vascular branches (9). Specifically, patients receiving UNSES reported significantly milder low back pain as well as less severe radicular leg pain compared with patients with UBE throughout the short-term follow-up. In addition, the increase of C-reactive protein was lower and the length of hospital stay was shorter in the UNSES group, suggesting that the tissue trauma reaction was more mild, which was in line with the core demand of the minimally invasive surgery of “less trauma and faster recovery.” Notably, all patients received fully unified postoperative analgesia, anticoagulation, and rehabilitation schemes in this study; therefore, the intergroup differences in perioperative trauma indicators and medium-term functional scores are unlikely to be attributed to inconsistent perioperative intervention but mainly derived from the inherent structural differences between single-channel UNSES and dual-channel UBE techniques.

In terms of complications, the overall complication rate was 3.03% in the UNSES group compared with 8.33% in the UBE group, although the intergroup difference did not reach statistical significance. Adverse events presented distinct distribution patterns within this small cohort: dural sac tear and epidural hematoma were observed only in patients with UBE, while the sole complication in the UNSES cohort was surgical site infection. We provide clinically reasonable anatomical interpretations for this case distribution: the dual-channel setup of UBE requires two separate paraspinal dissection planes, which may cause scattered bleeding points and relatively great challenges for complete hemostasis (10, 11); drill manipulation also carries a risk of visual field offset that could potentially irritate the dura mater. In contrast, UNSES uses a single fixed working tract, with limited instrument-dura contact and less disruption of the paraspinal vasculature, which facilitates precise hemostasis during surgery (12). Notably, these mechanistic explanations are only speculative clinical reasoning for the limited adverse events captured in the present small sample. The low absolute count of complications in both arms prevents drawing robust conclusions about consistent intergroup differences in complication risk, and we cannot infer that UBE inherently carries a higher risk of dural tear or hematoma based solely on this dataset. The infection rate was comparable between the two groups, indicating that infectious complications mainly depend on a standardized aseptic technique and postoperative wound care rather than an endoscopic channel design. No suture-associated adverse events were recorded in either cohort, thus verifying the safety and feasibility of uniform manual annular repair for both minimally invasive endoscopic modalities (13). Larger prospective or multicenter studies with higher complication event volumes are needed to reliably identify whether genuine disparities in complication profiles exist between UNSES and UBE.

Both UNSES and UBE rely on endoscopic visualization to complete thorough nucleus pulposus decompression. While fully removing herniated disc tissue, both modalities preserve native spinal anatomical structures and spinal stability, avoiding extensive soft tissue destruction seen in traditional open discectomy and creating favorable conditions for early postoperative functional rehabilitation (14). Radiological assessment at 6 months revealed divergent AF closure rates between the two groups, alongside differences in short-term pain and functional scores, which stem from the inherent channel characteristics of each endoscopic technique. The technical differences in annular suturing between the two endoscopic modalities partly account for the divergent 6-month complete annulus fibrosus closure rates on MRI. UNSES relies on one single narrow tract that limits instrument mobility during stitching; miniaturized forceps and frequent lens adjustment slightly hinder precise matching of torn annular edges. In contrast, UBE's separated observation and working channels maintain a steady full visual field, allowing flexible rotation of suture needles and easier tight apposition of wide, irregular annular lacerations intraoperatively. Although both groups followed identical standardized suture material, stitch count, and intraoperative closure criteria, the superior operating space of UBE facilitates more complete fibrous tissue healing at the tear site in the medium term, which corresponds to the statistically higher complete AF closure rate observed in the UBE cohort at the 6-month follow-up.

### 4.1 Limitations and future research prospects

This study carries several inherent limitations. First, this single-center retrospective cohort lacked random surgical allocation. UNSES or UBE was selected according to preoperative anatomical imaging and clinical judgment instead of randomization. Although baseline clinical and imaging parameters (symptom duration, pain/ODI scores, neurological function, disc and annular tear morphology) were well balanced between the groups, unmeasured confounders could not be completely ruled out. To reduce bias, uniform perioperative analgesia, anticoagulation, antibiotics, and rehabilitation were applied to all patients, eliminating interference from inconsistent postoperative care. Postoperative MRI scans were double-blinded and independently assessed by two senior surgeons using unified AF healing criteria, which lowered subjective radiographic bias. Even with these controls, observed intergroup differences cannot be fully attributed to the technical features of the two endoscopy approaches. Second, every participant received standardized manual annular suture, with a non-suture control arm absent from this cohort. Hence, we cannot quantify the isolated long-term benefits of annular repair, including its potential to lower reherniation risk and slow intervertebral disc degeneration. Third, follow-up was restricted to 6 months, and therefore, long-term outcomes such as recurrent herniation and progressive disc height loss remain unassessed. Furthermore, total blood loss was calculated using the hemoglobin difference method; perioperative fluid-induced hemodilution may introduce mild bias when comparing bleeding volume between groups. In addition, the small overall sample size and low incidence of complications limit our ability to draw definitive comparative conclusions on adverse event profiles between the two surgical techniques.

Several directions are proposed for subsequent research to overcome the above drawbacks. Prospective randomized controlled trials are needed to balance baseline anatomical confounders and mitigate selection bias when comparing UNSES and UBE techniques. Further controlled trials within a single endoscopic modality should be designed to compare patients receiving AF suture vs. those without repair, which will help clarify the stand-alone therapeutic value of annular closure. In addition, longitudinal follow-up spanning 2–5 years should be implemented to capture long-term disc degenerative changes and the cumulative incidence of recurrent herniation.

### 4.2 Conclusion

Our 6-month retrospective analysis enrolled patients with single-segment lumbar disc herniation who all received identical manual annular suturing during endoscopic decompression. The two minimally invasive endoscopic approaches delivered comparable short-term clinical safety and symptom relief, yet obvious technical discrepancies were observed in perioperative trauma metrics and medium-term imaging outcomes. UNSES demonstrated advantages in shortening operative duration, reducing intraoperative blood loss, and suppressing postoperative inflammatory response, accompanied by persistently milder back and radicular pain across all follow-up time points. In contrast, patients treated with UBE obtained superior lumbar disability scores and slightly higher complete annulus fibrosus healing rates at the 6-month MRI assessment. Owing to the short half-year follow-up window of this cohort, we cannot draw reliable judgments on long-term risks such as recurrent herniation and progressive intervertebral disc degeneration. Large-sample prospective studies with at least 1–2 years of serial imaging and recurrence surveillance are warranted to further verify the long-term comparative merits of these two endoscopic techniques.

## Data Availability

The original contributions presented in the study are included in the article/Supplementary Material, further inquiries can be directed to the corresponding authors.
